# Missing images: autobiographical memory in Aphantasia and blindness

**DOI:** 10.3389/fcogn.2025.1644533

**Published:** 2025-09-11

**Authors:** Cornelia McCormick, Sven Lange

**Affiliations:** ^1^Department of Old Age Psychiatry and Cognitive Disorders, University Hospital Bonn, Bonn, Germany; ^2^German Center for Neurodegenerative Diseases (DZNE), Bonn, Germany

**Keywords:** scene construction, visual perception, hippocampus, mental imagery, neural networks

## Abstract

Mental visual imagery, especially the ability to construct naturalistic scenes seems central to vivid episodic autobiographical memory (AM). This mini review will first highlight the neural anatomy of different aspects of mental imagery, focusing on the roles of the hippocampus, ventromedial prefrontal cortex and posterior neocortex and the consequences of damage to these regions to AM. We will then contrast the consequences of missing images for AM in two special populations with no apparent brain damage: Congenital Aphantasia (i.e., lack of visual imagery) and congenital blindness (i.e., lack of visual perception). We propose that Aphantasia leads to impaired scene construction and reduced AM reliving. Despite limited evidence on AM in congenitally blind individuals, they seem to rely on auditory and tactile information to construct (scene) imagery, which in turn may support vivid AM reliving. The main findings here suggest that mental scene imagery, rather than visual encoding, is crucial for AM. This conclusion has far-reaching implications for understanding memory disorders, mental health, and a call to protect our imagination.

## 1 Introduction

Vivid mental imagery features as a central cornerstone in episodic autobiographical memory (AM; Tulving, 2002; Sheldon and Levine, 2013). Decades of neuroimaging and neuropsychological research have established the tight link between these mental images and our ability to remember past events, which shape our sense of self and identity (Tulving, 2002). AM is not only vital for envisioning the future (Addis et al., 2007), making complex decisions (Buckner, 2010), and showing compassion (Strikwerda-Brown et al., 2019), but its impairment is also associated with conditions such as neurodegenerative dementias (Strikwerda-Brown et al., 2019), temporal lobe epilepsy (St-Laurent et al., 2009; McCormick et al., 2018b), and limbic encephalitis (Miller et al., 2020), carrying severe personal and economic impacts. Despite this significance, the neural mechanisms underlying AM remain poorly understood. While much of the research focuses on the neural networks of AM and the consequences of brain damage, two special populations, those with congenital Aphantasia (diminished visual imagery) and congenital blindness (diminished visual perception), provide a valuable lens to examine the impact of missing images on episodic AM retrieval. This opinion piece will briefly recapitulate what is known about the connection between AM and mental imagery before focusing on these two populations, ultimately drawing conclusions and proposing new hypotheses about the importance of images for AM.

A defining feature of AM is its vivid, detail-rich reliving experience. Some individuals virtually “see the event unfold” in their mind's eye. Without this, AM appears vague and dim (Viskontas et al., 2000). Vivid mental imagery, especially in the visual domain seems therefore crucial for vivid AM (Greenberg and Knowlton, 2014). In line, visualization abilities predict the detailedness of an imagined event and the vividness of a memory (Greenberg and Knowlton, 2014; Conway and Pleydell-Pearce, 2000; Greenberg et al., 2005; Greenberg and Rubin, 2003). A key question is whether all kinds of mental imagery are important for episodic, detail-rich AM. Are individual episodic elements (e.g., visual detail—the redness of a dress, or emotional detail—the joy felt) important or is the mental model of a visuospatial scene (e.g., standing in front of a house door) crucial (Maguire and Mullally, 2013)? In favor for naturalistic scenes, AM vividness is strongly predicted by our ability to mentally construct naturalistic scenes (Clark and Maguire, 2020). Participants recall events more vividly when AM can unfold with the visuoperceptual scaffold of scene-cues, as opposed to people-cues (Robin et al., 2018). When only people- cues are used, participants automatically add visual scenes. Additionally, mind-wandering episodes contain for the vast majority naturalistic scene imagery (McCormick et al., 2018c). These findings form the basis of the scene construction theory (Maguire and Mullally, 2013), which proposes that naturalistic scenes are the building blocks for vivid AM. In contrast, specific deficits in mental imagery, such as color or face blindness (prosopagnosia), do not lead to dramatic AM deficits (Epstein et al., 1999; Kanwisher, 2000). Thus, some forms of mental imagery seem more important to AM than others. This differentiation is also supported by the fact that different forms of mental imagery are supported by different brain structures. Box 1 will focus on the contributions and interactions of the hippocampus, posterior neocortex, and ventromedial prefrontal cortex (vmPFC).

Box 1 **The anatomy of AM and mental imagery.**

Autobiographical memory (AM) is intricately tied to mental imagery and the construction of visuospatial scenes (Hassabis et al., 2007; Maguire and Mullally, 2013) relying on a shared neural network that includes the hippocampus, ventromedial prefrontal cortex (vmPFC), and posterior neocortex (Hassabis and Maguire, 2009; McCormick et al., 2015; Robin et al., 2019; Svoboda et al., 2006). Each contributing unique features to AM and scene construction.

**The hippocampus**

The hippocampus plays a central role in retrieving vivid, detail-rich memories (Scoville and Milner, 1957; Rosenbaum et al., 2008; Viskontas et al., 2000) and constructing naturalistic scenes (Hassabis et al., 2007; Maguire and Mullally, 2013; McCormick et al., 2018a; Bakermans et al., 2025; Angeli et al., 2025; Clark et al., 2022). While the anterior segment of the hippocampus seems more engaged during scene construction, its posterior segment may be more engaged during scene perception (Angeli et al., 2025; Zeidman and Maguire, 2016). Additionally, the pre-/parasubiculum subfields of the hippocampus seem especially engaged in constructing mental scenes (Dalton and Maguire, 2017; Dalton et al., 2018) and AM (Leelaarporn et al., 2024).

*Patients*. Autobiographical amnesia is the hallmark of hippocampal damage (Scoville and Milner, 1957; Rosenbaum et al., 2008; Viskontas et al., 2000; Miller et al., 2020). Additionally, patients with bilateral hippocampal damage exhibit impaired scene construction (Hassabis et al., 2007; McCormick et al., 2018c, 2017, 2016). Thus, in our model, the hippocampals' most critical contributions to AM are mental models of naturalistic scenes.

**The posterior neocortex**

The posterior neocortex is thought to contribute visuo-perceptual details to AM, with specialized regions such as the fusiform gyrus and parahippocampal place area processing specialized details, such as faces and places (Epstein et al., 1999; Kanwisher, 2000). Higher associative cortices, including the angular gyrus and precuneus, integrate sensory input to reconstruct visual details, demonstrating the close overlap between visual perception and mental imagery (Svoboda et al., 2006; Dijkstra et al., 2019).

*Patients*. Damage to the posterior neocortex typically result in selective perceptual deficits, such as prosopagnosia (Kanwisher, 2000), and sometimes to impaired AM (Greenberg et al., 2005; Rubin and Greenberg, 1998; Ramirez-Bermudez et al., 2024).

**The ventromedial prefrontal cortex**

Traditionally, the vmPFC has been linked to roles such as emotion regulation (Bechara et al., 2000), decision-making (Damasio, 1996), and moral reasoning (Koenigs et al., 2007), but also memory and learning (Gilboa and Marlatte, 2017). We suggested, the vmPFC initiates and elaborates temporally extended mental scenarios, interacting with the hippocampus and posterior neocortex to integrate snapshots into coherent narratives (McCormick et al., 2018a; Barry et al., 2019; McCormick et al., 2020; Monk et al., 2021).

*Patients*. vmPFC-damaged patients show AM and scene construction deficits, as well as a reduced ability to initiate endogenous mental scenarios (Bertossi and Ciaramelli, 2016; Bertossi et al., 2016a,b). Nonetheless, the construction of individual scenes maybe intact (Kurczek et al., 2015; De Luca et al., 2019). These differences indicate the vmPFC's role in integrating successive scenes into extended narratives.

**Summary**

**Figure:**
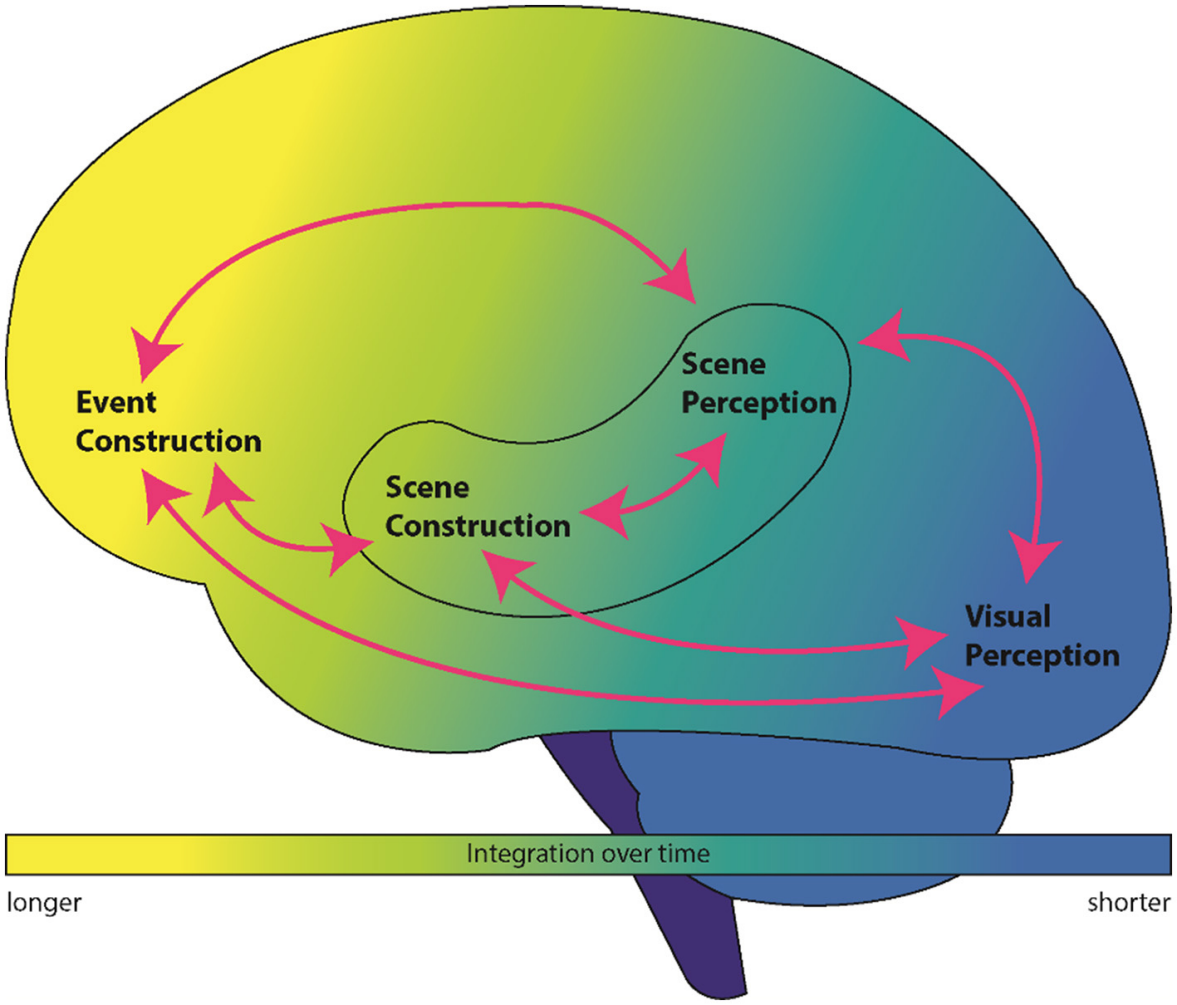
This figure shows the contributions of the vmPFC, namely event construction, the hippocampus, scene construction and perception, and the posterior neocortex, visual perception to AM. Damage to any of these regions can result in impaired ability to construct mental events, leading to AM deficits. Thus, hampering with our inner images, especially in forms of naturalistic scenes, seems to be detrimental to vivid AM recall. *The arrows signify strong functional connectivity. The color gradient symbolizes the transition from fine-grain visual details (blue) to extended, multimodal autobiographical memories (yellow)*.

In conclusion, constructing vivid, imagery-rich mental events, like episodic AM, relies on an intricate neural machinery that allows us to mentally “see” events unfold upon a visuospatial stage. Brain damage to any of these regions can impair our mind's eye, potentially leading to AM deficits and significant cognitive and emotional changes. Research suggests the visual system drives the construction of vivid mental events, especially in sighted people. Thus, a major gap in our understanding is whether inner visual scene imagery depends on visual experience. The following will synthesize evidence from two special populations: people with Aphantasia, who lack inner visual images, and people blind from birth, who lack visual perceptual experience.

## 2 Autobiographical memory and scene construction in Aphantasia

Aphantasia is a neuropsychological normvariante characterized by a significant reduction or complete lack of voluntary sensory imagery (Monzel et al., 2022) with its neural underpinnings still being debated (Blomkvist and Marks, 2023; Pearson, 2019). Typically, Aphantasia is identified by low subjective ratings on the Vividness of Visual Imagery Questionnaire (VVIQ; Marks, 1973) and it is associated with psychophysiological changes, such as reduced imagery-induced pupil contraction (Kay et al., 2022) and diminished imagery-induced priming effects (Keogh and Pearson, 2018; Monzel et al., 2021).

In terms of AM, several studies have reported convergent evidence that people with Aphantasia recall fewer AM details compared to controls (Monzel et al., 2024; Dawes et al., 2020; Milton et al., 2021; Zeman et al., 2020; Dawes et al., 2022). This effect was found for recent and remote AM (Monzel et al., 2024; Milton et al., 2021) and consistent over multiple sensory details, including visual (Dawes et al., 2022), time, place, and emotion (Monzel et al., 2024). Thus, the AM deficit in Aphantasia is not only confined to missing visual details, but rather to a global reduction in episodic details. Albeit marked differences between healthy people with Aphantasia and individuals with pathological hippocampal damage, this profile of AM deficits resembles this found in individuals with hippocampal damage (Rosenbaum et al., 2008). Memories of people with Aphantasia also tend to be less emotional and are reported with less confidence (Monzel et al., 2024; Dawes et al., 2022; Wicken et al., 2021). A recent neuroimaging study indicated that Aphantasia is associated with decreased hippocampal activity and increased visual-perceptual cortex activity during AM retrieval (Monzel et al., 2024). In controls, stronger connectivity between the hippocampus and visual-perceptual cortex was linked to better visualization skills, however, in Aphantasia, this connectivity correlated with worse visualization skills. Other recent neuroimaging studies also suspect the early visual cortices and their neocortical connectivity to play a crucial part in the neural underpinnings of Aphantasia (Cabbai et al., 2024; Montabes de la Cruz et al., 2024; Chang et al., 2025). For example, decoding of perceptual content from early visual cortex was less in Aphantasia (Chang et al., 2025). Together, these findings suggest that mental imagery construction is crucial for vivid AM retrieval and is supported by hippocampus-visual cortex connectivity.

In addition to the significant differences in the subjective relieving of AM, people with Aphantasia also tend to report less details if they are asked to conjure up atemporal, novel scenes and future scenarios (Milton et al., 2021; Dawes et al., 2022). These findings are reflected by their low ratings on the VVIQ, which requires individuals to construct vivid mental scenes (Bainbridge et al., 2021), but also employing more extended interview techniques (Milton et al., 2021). Together, the recent evidence on Aphantasia suggests that, despite an intact visual system (Cabbai et al., 2024; Chang et al., 2025) and no gross brain pathology (Milton et al., 2021), the lack of vivid mental imagery leads to profound deficits in recalling episodic AM and constructing mental models of scenes. Interpreting this constellation by referring to Box 1, it seems likely that the neural underpinnings of Aphantasia lie especially in the visual cortices and their communication with the hippocampus. This conclusion leads to the imminent question whether people who are blind from birth and thus, lack visual perception, also display these AM alterations.

## 3 Autobiographical memory and scene construction in blind people

In contrast to people with Aphantasia, people who are blind due to ophthalmological reasons (see Box 1 for the impact on AM due to CNS-damage to the visual system) cannot encode the world visually, which hampers their ability to encode naturalistic scenes. To date, there are only a handful of heterogeneous studies examining AM in blind individuals. We identified seven studies that examined AM in blind people (Cornell Karnekull et al., 2020; Pring and Goddard, 2004; Tekcan et al., 2015; Eardley and Pring, 2006; Güneş-Acar and Tekcan, 2024; Goddard and Pring, 2001; Ally et al., 2013). These were behavioral studies using different AM tasks, mostly with small sample sizes and including blind participants with varying onsets and severity of blindness.

One important caveat in interpreting these findings is that much evidence indicates that the function of the visual cortex develops postnatally based on visual input (Wiesel and Hubel, 1965). There seems to be a critical period in development, suggesting that congenital blindness has a profoundly different impact on cortical development than becoming blind later in life (Hooks and Chen, 2007). Thus, it likely makes a difference to AM whether people are congenitally blind or became blind after some years of visual experience, and whether they are totally blind or still perceive visual/scenic details. Specifically, remaining visual perception of sky and ground could still enable scene perception and facilitate scene construction.

The little coherence in the findings suggests that blind people may have relatively subtle difficulties recalling specific events. Five out of the seven studies reported that blind individuals needed more prompting to retrieve specific memories (Tekcan et al., 2015; Eardley and Pring, 2006; Güneş-Acar and Tekcan, 2024; Goddard and Pring, 2001; Ogden and Barker, 2001). These results were consistent despite different cues (auditory sounds, odors, concrete, and abstract words). One study did not find this effect (Cornell Karnekull et al., 2020), and a case study reported even heightened AM retrieval access in a congenitally blind person (Ally et al., 2013). Importantly, most studies reported measures of episodicity, reliving experience, and detail-richness, with no group differences. These findings indicate that the feeling of re-experience seems to be as vivid as that of sighted people. Blind people seem to report more auditory and non-episodic details than sighted controls (Tekcan et al., 2015; Güneş-Acar and Tekcan, 2024) and rate their memories as more important and temporally extended (Güneş-Acar and Tekcan, 2024). A case study of a 20-year old man who was born prematurely and suffered from retinopathy of prematurity reported superior AM with heightened accuracy and reliving of auditory and tactile details. This patient had reduced hippocampal volume but increased amygdala volume and strong fMRI resting state connectivity to the right hippocampus (Ally et al., 2013).

This scare literature reveals a major knowledge gap in our understanding of AM and its neural signature in blind people; and whether it makes a different for AM, if a person is blind from birth or late blind. From the little evidence there is, the vivid re-experience seems to resemble that of sighted individuals. These finding mesh well with evidence that their episodic memory *per se* appears intact (Roder et al., 1999; Amedi et al., 2003; Raz et al., 2005), and in some cases, when auditory cues are presented, even superior to that of sighted controls. Much more research has been done in spatial navigation and mental imagery in blind people. While a comprehensive review of this literature is beyond the scope of this opinion piece, in the next section, we will briefly explore these topics with the question in mind whether it is likely that people who are blind from birth have the ability to construct mental models of naturalistic scenes.

## 4 Mental scene imagery in blind people

To our knowledge, there are no studies specifically examining the construction of mental models of scenes in congenitally blind people. Thus, we approach this topic by first reviewing, mental imagery and its neural correlates, and then spatial representations and their hippocampal reflections.

### 4.1 Mental imagery and the posterior neocortex in congenitally blind people

There is good evidence that mental imagery in many perceptual domains, especially tactile and auditory imagery, of congenitally blind people remains intact, sometimes superior to that of sighted controls (Bleau et al., 2022; Chebat et al., 2020; Renzi et al., 2013). In the visuospatial domain, however, blind people lack accuracy and vividness. For example, objects that cannot be experienced through touch (e.g., wild animals) are rated lower in vividness by blind individuals compared to objects that can be touched (e.g., tools; Tian et al., 2024). Their visual concepts tend to be more abstract and semantic (Cattaneo et al., 2008; Cornoldi et al., 1993) and reliant on previous tactile exploration of the objects or descriptions provided by others (Lambert et al., 2004; Striem-Amit et al., 2018; Xu et al., 2023). An unresolved debate in this context is whether the mental representations of congenitally or early blind people are more “propositional” (i.e., based on abstract, language- mediated concepts) or analogical (vision-like). De Volder et al. (2001) introduced the term “shape-knowledge” (visual semantics) to describe imagery abilities in congenitally blind individuals. According to this view, auditory and tactile senses partially create vision in the brain by acting as a natural substitute for lost visual input during brain maturation, enabling the development of specific visual functions. Further, non-visual sensory modalities like auditory, haptic/tactile, and olfactory imagery enable neuroplastic adaptation of the occipitotemporal cortex in the absence of early visual stimulation (Xu et al., 2023; De Volder et al., 2001; Vetter et al., 2020). Specifically, visual imagery in sighted individuals and tactile imagery in congenitally blind people recruit the same brain areas, such as the superior occipital and visual association areas (Lambert et al., 2004). In addition, support for this model of cross-modal neuroplasticity of the “blind visual cortex” comes from its involvement in episodic memory (Raz et al., 2005), language (Sadato et al., 1996), audition (Vetter et al., 2020), and haptic (Amedi et al., 2010) processing. Thus, similar to sighted people, the construction of mental images in blind individuals seems to rely on the activation of occipital areas (Xu et al., 2023; De Volder et al., 2001; Vetter et al., 2020; Amedi et al., 2004). Thus, whereas visual mental imagery may be altered, the mental representations of many types of imagery remains intact and reliant on the posterior neocortex, similar to that of sighted people.

### 4.2 Spatial representation and the hippocampus in congenitally blind people

**Figure 1:**
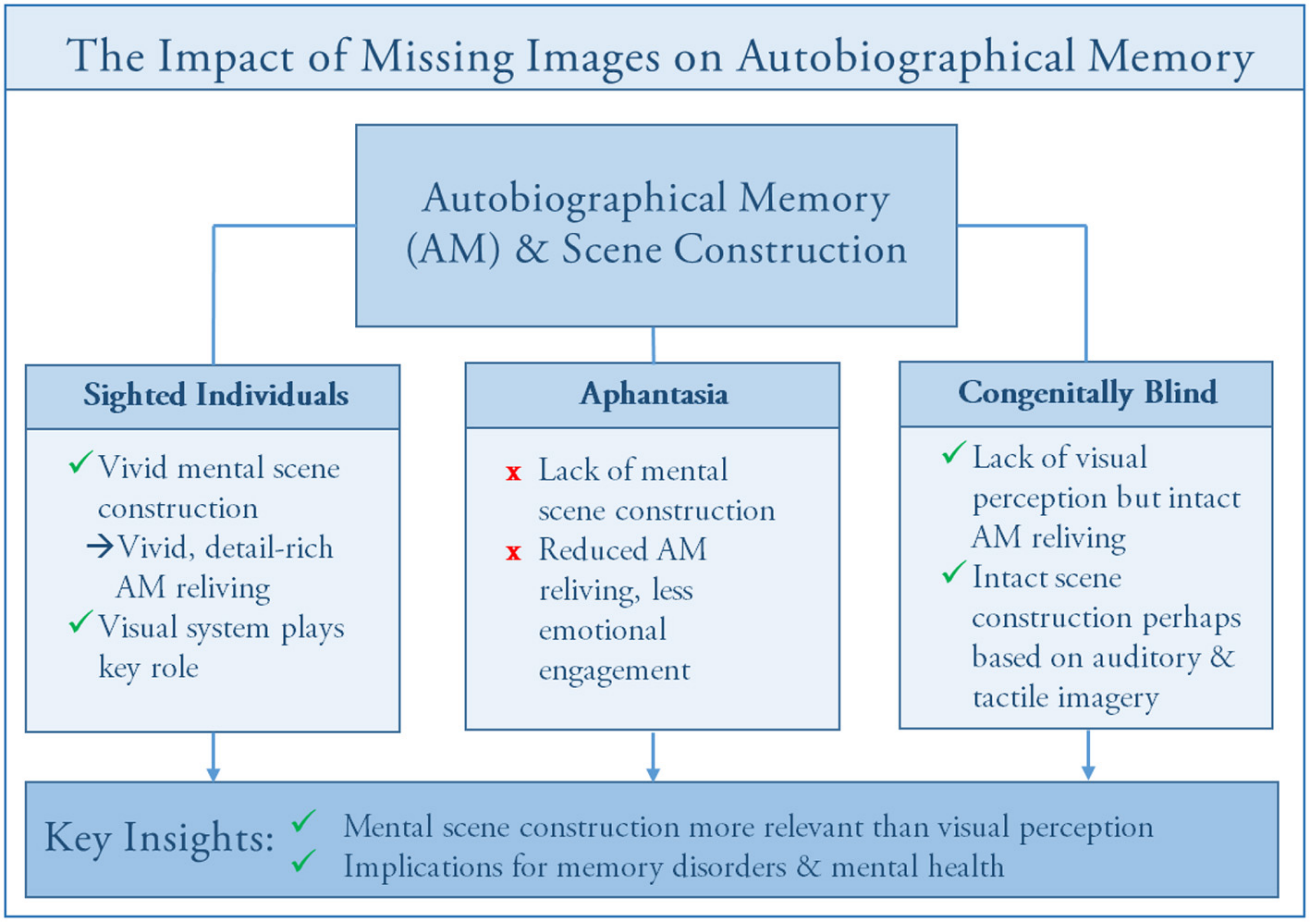
The impact of missing images on AM. This figure illustrates the main conclusions that the lack of visual perception (congenital blindness) can be compensated as along as if the construction of mental scenes is intact. Otherwise, as due to brain damage or Aphantasia, AM reliving is deficient.

A recent meta-analysis examined neural structures supporting spatial navigation and spatial representation in congenitally blind people. They included 31 studies in an activation likelihood estimation (ALE) analysis and reported significant overlap between the neural structures supporting spatial cognition in blind and sighted people (Bleau et al., 2022). Although hippocampal volume has been found to be reduced in many congenital blindness [(Chebat et al., 2020; Pan et al., 2021), but see (Fortin et al., 2008)] the hippocampus was engaged during spatial navigation tasks (Bleau et al., 2022). Similar conclusions were drawn in an earlier review (Chebat et al., 2020) in which the authors report that congenitally blind people can re-interpret auditory and tactile information to compensate for the lack of vision in order navigate and represent space equally well to sighted people. Accordingly, blind people are able to avoid obstacles, remember locations, integrate paths and generate cognitive maps. Whereas, the acquisition of spatial representations seems to take longer and neural differences do exist (Sigismondi et al., 2024; Pasqualotto and Newell, 2007), in general, congenital blindness does not lead to a spatial navigational deficit and a deficit in the mental representation of space. Thus, it seems likely that the construction of naturalistic scenes, even if they are represented auditorily [so called soundscapes (Dong and Karmann, 2024)] is intact in congenitally blind people (Figure 1).

## 5 Conclusions

Episodic AM is crucial for shaping our sense of self, envisioning the future, and showing compassion. Impairments in AM, seen in conditions like dementia and epilepsy, highlight the importance of understanding how these memories are encoded and retrieved. The ability to construct naturalistic scenes appears to be a key driver of the vividness of AM. Despite this strong link, there is a knowledge gap in understanding the impact of missing images to AM. This review highlights a stark difference between the episodic AM recall of two special populations with no gross brain pathology and for both of which images are missing for different reasons. Insights from Aphantasia (i.e., lack of mental imagery) show a significant deficit in constructing mental scenes and with that, reduced reliving of AM. In contrast, limited evidence in individuals with congenital blindness (i.e., lack of visual perception) suggest a seemingly intact feeling of AM reliving. We conclude first that more research is needed to explore AM and scene construction in blind people, and second, that the construction of mental models of scenes allow for a rich and vivid re-experience of AM. In fact, the perception of visual images seems to be of lesser importance than the internal construction of scenes. This conclusion has significant implications for diagnosing and treating memory disorders, enhancing mental health, and understanding the brain's adaptability in sensory deficits.

## 6 Outstanding questions

- **Prioritize research into AM in blind individuals:** Studies on AM in blind populations are urgently needed. Given the critical period for the neurodevelopment of the visual cortex, it is essential to differentiate between individuals who are congenitally blind and those who lost their sight later in life.
- **Investigate the neural correlates of AM in blindness:** A deeper understanding of the neural mechanisms underlying AM in blind individuals is crucial. Does their AM re- experience rely on the hippocampus and its connectivity in a manner comparable to sighted individuals, or are alternative neural pathways recruited?
- **Uncover blind individuals' capacity for scene construction:** While mental imagery and spatial cognition have been studied in blind individuals, little is known about their ability to construct scenes and simulate future scenarios. What are the neural bases of these fundamental cognitive processes in the absence of visual experience?
- **Conduct comparative studies on missing imagery:** A direct comparison between individuals with Aphantasia and those who are congenitally blind could provide transformative insights into how missing mental images influence AM and scene construction.
